# The Multiple Functions of B Cells in Chronic HBV Infection

**DOI:** 10.3389/fimmu.2020.582292

**Published:** 2020-12-14

**Authors:** Ying Cai, Wenwei Yin

**Affiliations:** Key Laboratory of Molecular Biology for Infectious Diseases (Ministry of Education), Department of Infectious Diseases, Institute for Viral Hepatitis, The Second Affiliated Hospital, Chongqing Medical University, Chongqing, China

**Keywords:** B cell, hepatitis B virus, antibody production, antigen presentation, immune regulation, immunotherapy

## Abstract

Chronic hepatitis B virus (HBV) infection is one of the main causes of liver diseases, of which the natural history and clinical outcomes are associated with the role of B cells. As humoral immune cells, B cells play a critical role in the process of anti-HBV antibody production. In addition, some studies have also characterized other B cell subsets involved in antigen presentation and regulating the immune response beyond antibody secretion. However, not all B cell subsets play a positive role in the immune response to chronic HBV infection, and various B cell subsets jointly mediate persistent HBV infection, tolerance, and liver damage. Thus, we further sought to elucidate the multiple functions of B cells to gain novel insight into the understanding of chronic hepatitis B (CHB) pathogenesis. We also reviewed the current immunotherapies targeting B cells to explore novel therapeutic interventions for the treatment of chronic HBV infection.

## Introduction

Hepatitis B virus (HBV) is a serious global public health problem and major cause of chronic hepatitis, cirrhosis, and hepatocellular carcinoma (HCC). It is estimated that more than 250 million people worldwide are chronically infected with HBV (1), and there are approximately one million deaths attributed to HBV-related complications [*e.g.*, cirrhosis and hepatocellular carcinoma (HCC)] each year (2).

Following HBV infection, the risk of progression to chronicity is age-dependent. Approximately 90% of those who acquire HBV perinatally or in early childhood will develop chronic infection, whereas only 2–6% of people who are infected with hepatitis B as adults become chronically infected (3). In contrast, HCV infection progresses to a chronic persistent infection in 60–80% of infected adults (3). Generally, the natural course of chronic HBV infection can be divided into four chronological phases based on the virus–host interactions (4). The immune-tolerant phase is characterized by the active replication of HBV, HBV e antigen (HBeAg) positivity, and normal alanine aminotransferase (ALT) levels. In the immune clearance phase, HBeAg-positive patients have elevated serum ALT levels and fluctuating HBV-DNA levels. The third stage represents the inactive carrier state, in which patients clear HBeAg and develop the corresponding antibody to HBeAg (HBeAg seroconversion), with the remission of liver disease. In addition, approximately 20−30% of individuals in the inactive carrier state may experience a viral relapse and enter the reactivation phase during follow-up (4).

The pathology of hepatitis B is diverse and reflects the natural course of the disease. Acute hepatitis B is characterized by lobular disarray with swollen hepatocytes, numerous apoptotic bodies, hyperplastic Kupffer cells, and lymphocyte-predominant lobular and portal inflammation (5). In chronic hepatitis B, there is a varying degree of predominantly lymphocytic portal inflammation with interface hepatitis and spotty lobular inflammation. Inflammation is less pronounced in the immune-tolerant and inactive carrier phases, but is prominent in the immunoreactive phase. Unlike hepatitis C, chronic hepatitis B is usually not associated with lymphoid aggregates, duct (Poulsen) lesions, or steatosis (5). A typical histological feature of chronic hepatitis B is the ‘ground-glass hepatocyte’, which is due to accumulation of hepatitis B surface antigen (HBsAg) within the endoplasmic reticulum (ER) (3).

The complex interactions between HBV and the host immune system drive the process of chronic HBV infection, in which the adaptive immune system plays an important role in inducing an HBV-specific immune response. Because CHB patients are typically characterized by the dysfunction and exhaustion of HBV-specific CD4+ and CD8+ T cells (6, 7), specific T cell-mediated immune responses have become the focus of attention in HBV infection and clearance. At present, there are a substantial number of studies that have described T cell defects during a persistent HBV infection, which are mainly characterized by the sustained expression of multiple inhibitory receptors, poor effector cytotoxic activity, and impaired cytokine production (7–9). However, the role of B cells in HBV infection is often overlooked. In studies related to chronic HBV infection, there has been a reported increase in the percentage of B lymphocytes in the peripheral blood (10). In addition, B lymphocytes had infiltrated the liver (approximately 15% of the inflammatory infiltration) (11) and were clustered in the portal areas or were single cells within the lobule, which are related to liver inflammation and fibrosis (12). Therefore, it is interesting to determine whether, and how, B cells are involved in chronic HBV infection.

Knowledge of the role of B cells has consistently been primarily focused on antibody secretion during chronic HBV infection; however, as an important part of the adaptive immune response, various immune functions of B cells (*e.g.*, antibody secretion, antigen presentation, and immune regulation) jointly mediate persistent HBV infection, tolerance, and liver damage. Therefore, attention should be paid to comprehensively understand the role of B cells in HBV infection, which is conducive to the prevention, monitoring, and treatment of HBV infection. This review describes these three aspects of B cell function during chronic HBV infection and explores the role of B cells in liver injury and immune tolerance during chronic HBV infection (**Table 1**). Finally, we discuss the directions of future development of B cell immunotherapy in clinical practice.

**Table 1:** The potential functions of B cells in chronic HBV infection.

Potential function	Mediators	Mechanism	Outcome	References
**Antigen presentation**	MHC-I molecular	Cross-present HBcAg on MHC-I to specific CD8+ T cells and induce CTL cytotoxicity	Increase apoptosis of HBcAg-involved cells	(13)
		Cross-present HBsAg on MHC-I to specific CD8+ T cells and induce CTL cytotoxcity	Increase apoptosis of HBsAg-involved cells	(14)
	MHC-II molecular	Present HBcAg on MHC-II to specific CD4+ T cells	Promote anti-HBc CD4+ T cell response	(15)
**Antibody production**	Self-antibodies	Induce an auto-reaction *via* self-antibodies	Impair liver tissue	(16)
	Anti-HBc antibodies	Prime immune complex formation, complement activation *via* classic pathway and mediate CDC	Increase HBV-infected hepatocyte lysis	(17–19)
	Anti-HBs antibodies	Produce anti-HBs antibodies to bind HBsAg, and block HBV entry and replication	Reduce HBV spread	(20, 21)
		Initiate immune complex formation, further recruit NK cells, and mediate ADCC	Increase apoptosis of HBV-infected hepatocytes	(17, 22)
		Initiate immune complex formation, further recruit Kupffer cells, and mediate ADCP	Promote HBV clearance	(23)
		Participate in immune complex formation and DC binding	Induce T cell priming	(24)
**Immune regulation**	IL-10	Inhibit effector T cells and enhance regulatory T cell function	Promote immune tolerance	(25, 26)
	IL-35	Inhibit the proliferation of effector T cells	Interfere with cellular immune responses	(27, 28)
	IL-6	1. Hinder HBV entry into hepatocytes and promote cccDNA decay to play a non-cytolytic antiviral activity 2. Support effector CD4+ T cell response	1. Reduce HBV infection and decrease HBV persistence 2. Increase effector CD4+ T cell responses	(29–31) (32, 33)
	IFN-γ, TNF-α	1. Induce cccDNA decay and then play a non-cytolytic antiviral activity 2. Influence the development and responses of CD4+ T cells	1. Decrease HBV persistence 2. Promote effector CD4+ T cell responses	(34) (32, 33)

ADCC, antibody-dependent cellular cytotoxicity; ADCP, antibody-dependent cellular phagocytosis; CDC, compliment-dependent cytotoxicity; MHC-I, major histocompatibility complex class I; MHC-II, major histocompatibility complex class II; DCs, dendritic cells.

## Antibody Production Function of B Cells

The early knowledge of HBV-specific B cells is primarily derived from the detection of serum antibodies that have important clinical implications. Antibodies against different HBV protein components, especially the envelope antigens (HBsAg) and nucleocapsid antigens (HBeAg and HBcAg), could be applied to the diagnosis and prediction of HBV infection (35). Anti-HBc IgM only appears during an acute HBV infection and severe exacerbation of chronic infection, whereas anti-HBc IgG is found throughout the prior, ongoing, and even occult HBV infection period (36). Quantitative serum anti-HBc levels may reflect the strength of the host adaptive anti-HBV immune activity (37, 38), and thus may serve as a predictor of HBeAg reversal following treatment with peg interferon or nucleos(t)ide analogs (NUCs) in CHB patients (39–41). Anti-HBe appears later than anti-HBc, and a high level of anti-HBe antibodies often predicts a better outcome. Immunity to HBV infection is associated with the secretion of protective anti-HBs antibodies, which represent recovery from an acute HBV infection or acquired immunity through HBV vaccination (36). In general, clinical significance exists between the various antibodies produced by HBV-specific B cells, which suggests that the function of HBV-specific antibody secretion by B cells is an important humoral immune response in HBV infection.

**Figure 1:**
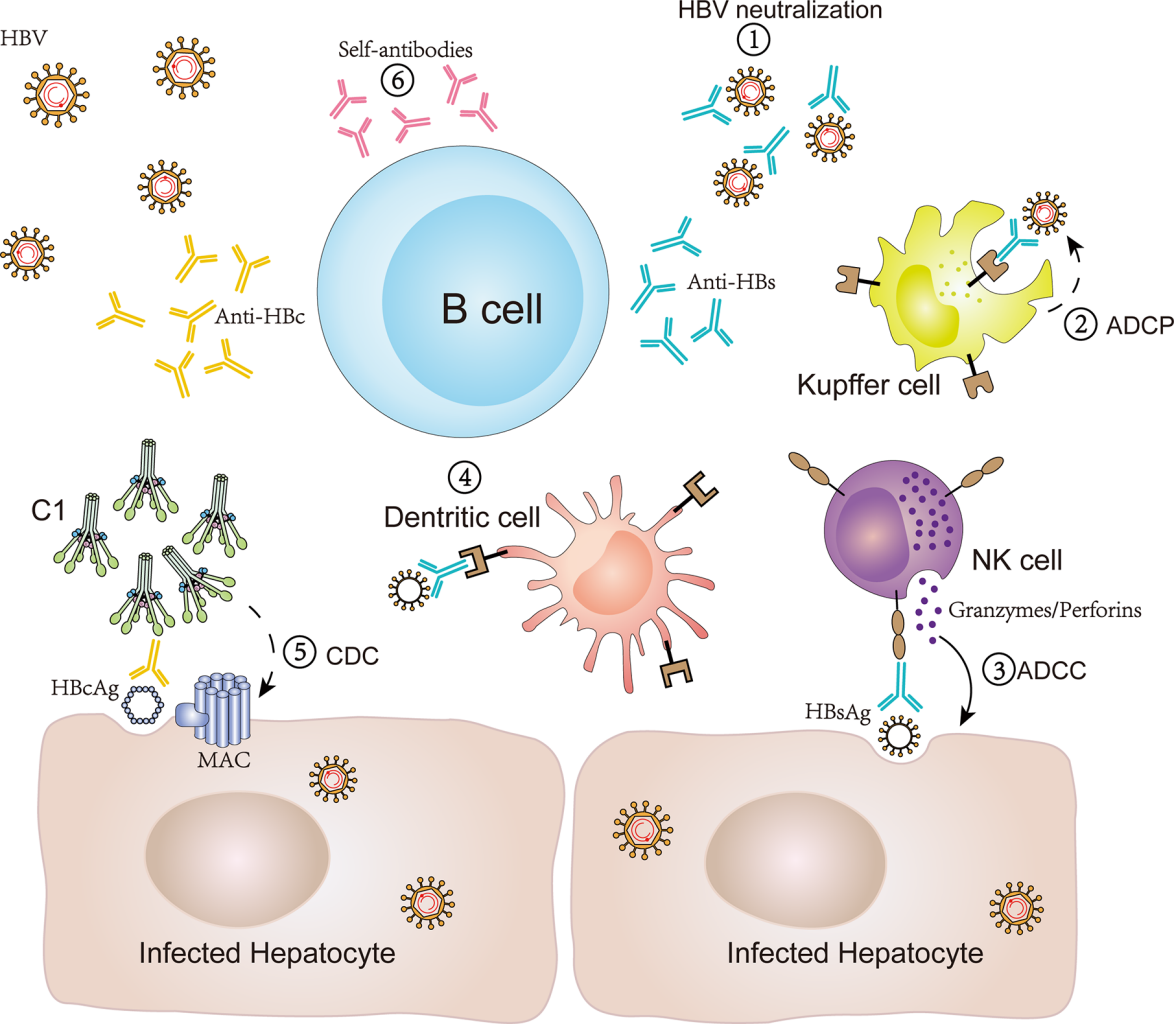
Antibody production by B cells and antibody-mediated immune response in CHB infection. B cells can produce antibodies, such as anti-HBs, anti-HBc and self-antibodies, and subsequently mediate an antiviral immune response or self-reaction *via* various potential mechanisms in CHB infection. (1) Anti-HBs antibodies bind to HBsAg to block viral entry and replication; (2) anti-HBs antibodies bind HBsAg and induce cellular phagocytosis of Kupffer cells to consume HBV (ADCP); (3) anti-HBs antibodies bind HBsAg and induce the release of perforin/granzyme in NK cells to eliminate HBV-infected hepatocytes (ADCC); (4) anti-HBs antibodies participate in forming immune complexes and bind to dendritic cells to induce a T cell response; (5) anti-HBc IgG binds HBcAg to induce hepatocyte lysis *via* the classical complement activation pathway initiating from C1 (CDC); (6) self-antibodies participate in an autoimmune reaction to aggravate liver inflammation. ADCC, antibody-dependent cellular cytotoxicity; ADCP, antibody-dependent cellular phagocytosis; CDC, compliment-dependent cytotoxicity.

In order to research the humoral immune response of HBsAg-specific B cells in CHB patients, two studies have used recombinant fluorochrome-labeled HBsAg as “bait” to analyze the frequency, phenotype, and function of such specific B cells in the blood (32, 42). It was found that HBsAg-specific B cells existed at a low frequency in blood of CHB patients and contained antiviral potential. However, the cellular phenotype was similar to CD21− CD27− atypical memory B cells (atMBCs), which express high levels of inhibitory receptors, such as programmed cell death receptor-1 (PD-1). Moreover, HBsAg-specific B cells isolated from HBV-infected patients could not efficiently expand and mature into antibody-secreting cells *in vitro*, whereas a PD-1 blockade or the addition of IL-2, IL-21, and CD40L may partially restore the function of HBsAg-specific B cells (32, 42, 43). Since anti-HBs antibodies can function as protective neutralizing antibodies to block HBV entry or replication, it can be inferred that the dysfunction of anti-HBs secretion by B cells is beneficial for the maintenance of high levels of HBsAg and hinders HBV clearance. Indeed, apart from antigen neutralization (20, 21), there are other potential effector functions of anti-HBs antibodies during chronic HBV infection. Based on the discovery of cytoplasmic and membranous HBsAg, anti-HBs IgG might bind HBsAg and induce antibody-dependent cellular cytotoxicity (ADCC) to deplete HBV-infected hepatocytes (17, 22). In theory, anti-HBs antibodies can also bind HBsAg and exert antibody-dependent cellular phagocytosis (ADCP) to consume HBV (23). During the HBV vaccine design process, anti-HBs antibodies are utilized to participate in the formation of immune complexes and bind to dendritic cells (DC), which further induce a T cell response (24). Therefore, there are multiple pathways mediated by anti-HBs antibodies against HBV infection (**Figure 1**). However, defects in HBsAg-specific B cells in the secretion of anti-HBs antibodies might promote a persistent HBV infection.

In subsequent studies that have compared HBsAg-specific B cells, the frequency of HBcAg-specific B cells was higher, with primarily an IgG+ classical memory B cells (cMBCs) phenotype in the peripheral blood (44). In contrast to HBsAg-specific B cells, HBcAg-specific B cells were able to expand and mature into antibody-secreting B cells *in vitro*, which is beneficial to sustaining a large amount of anti-HBc antibodies in CHB patients (44). However, the presence of anti-HBc antibodies has been shown to play a destructive role in hepatocyte activity and HBV clearance (45–47). Specifically, anti-HBc antibodies can promote the formation of immune complexes in the liver to initiate complement activation and induce massive liver necrosis, which have been confirmed in HBV-associated acute liver failure (ALF) (18, 19).

B cell-mediated humoral immune responses are regulated by T follicular helper (TFH) cells (48). It has been demonstrated that the frequency and phenotype of cTFH cells in CHB patients were altered following infection, and were relevant to clinical parameters, which indicates that cTFH cells may be involved in the immune response against HBV (48). In follow-up studies, there was further evidence that cTFH cells regulate the humoral immune response against envelope proteins and nucleocapsids. During chronic HBV infection, cTFH cells play an important role in the seroconversion of HBeAg (49), in which the secretion of IL-21 may be critical (50, 51). Similarly, HBsAg is a strict T cell-dependent antigen, and the production of anti-HBs antibodies or the seroconversion of HBsAg also requires the aid of TFH cells (52). However, during chronic HBV infection, there are two factors that inhibit the role of TFH cells in promoting B cells to produce anti-HBs antibodies. Specifically, it is plausible that the expression of HBcAg alone or excessive CD40L from activated TFH cells results in the expression of inhibitory receptors (FcRLs and PD-1) on B cells and the accumulation of more atypical memory B cells (53). Moreover, the suppression of HBsAg-specific TFH cells mediated by T regulatory (Treg) cells and follicular regulatory T (TFR) cells is associated with an impairment of HBsAg-specific B cell responses (54–56). Wang et al. found that the response of cTFH cells against HBsAg was blocked by Treg cells in mice with persistent HBV infection, whereas the depletion of Treg cells could restore the response (54). As a subset of Treg cells, CD4+Foxp3− type 1 regulatory T (Tr1)-like cells in the liver migrated to the draining lymph node (DLN) and also suppressed germinal center (GC) formation and anti-HBs antibody production (55). Furthermore, Foxp3+ TFR cells derived from natural Treg cells have many of the same properties as Tregs (57, 58), in which the increased circulating TFR (cTFR)-like cells might similarly impair cTFH cells and participate in chronic HBV infection (56). However, in contrast to HBsAg, HBcAg with high immunogenicity could induce both T cell-dependent and T cell-independent immune responses (59). According to the detected antigenic determinants of T cells, HBcAg-specific helper T (Th) cells were found to help B cells produce anti-HBc antibodies (60). At the same time, without the help of T cells, a population of human naive B cells were found to directly bind HBcAg and were subsequently activated to secrete HBcAg-binding IgM (61).

Apart from the abnormal regulation of TFH cells, the poor differentiation of specific memory B cells into anti-HBs-secreting plasma cells also further affected the humoral immune response of B cells in CHB patients. In humoral immune responses, B cells can differentiate through two distinct pathways. On the one hand, B cells can differentiate to form extrafollicular plasmablasts for rapid antibody production and early protective immune responses. On the other hand, activated B cells can differentiate into plasma cells in germinal centers and subsequently secrete high-affinity antibodies, which confer long-lasting protection from re-infection (62, 63). However, the differentiation of plasmablasts and anti-IgG antibody production may be partially hindered in CHB infection (64). In addition, according to the initial ELISpot examination of plasma cell formation, there were fewer global B cells and plasma cells isolated from the peripheral blood of CHB patients compared with the vaccinated controls (65, 66). One reason for this observation may be the enrichment of atMBCs in the liver and functional impairment of atMBCs’ differentiation into antibody-secreting plasma cells (32, 43). Another reason might be the enrichment of these virus-specific B cells in the liver of patients, which is not conducive to migration to the bone marrow and formation of long-lived plasma cells (32).

As with many chronic diseases, racial/ethnic disparities are seen in HBV infection. The overall prevalence of chronic HBV infection in the US was 2.74% in Asians, 0.6% in African-Americans, 0.06% in Hispanics, and 0.15% in Caucasians (67, 68). Ethnicity influences the natural history and immune responses during a chronic HBV infection. According to the Hepatitis B Research Network (HBRN) database, Asians with chronic HBV infection were more likely to have longer duration of infection and higher HBV DNA level compared with African-Americans and Caucasians, and proportionally more Caucasians had an increased ALT level more than two times the upper limit of normal (ULN) compared with Asians and African-Americans (69). Asian ethnicity was associated with lower rates of anti-HBe seroconversion in children born in endemic countries compared to other ethnicities (70, 71). Caucasian ethnicity was associated with an increased chance of HBsAg loss following nucleos(t)ide analogue-induced HBeAg seroconversion (72). Production of anti-HBs or anti-HBe antibody is helper T cell dependent. CD4+T cells recognize different epitopes within the HBsAg or HBeAg molecule that can be presented by certain major histocompatibility complex (MHC) class II antigens. Polymorphisms of MHC class II genes have been shown to be associated with HBV persistence, seroclearance, seroconversion, and disease progression, but only in patients with a certain ethnic background. For example, the HLA-DR polymorphism rs 9277535 (550 A/G) associated most significantly with chronic hepatitis B and its outcomes in Asian, but not in African-American or Caucasian patients (73). Differences in antibody production may contribute to the racial/ethnic differences observed in HBV prevalence and more work needs to be done to clarify the genetic and immunologic basis of the development of HBV infection stratified by race/ethnicity.

In summary, HBV-specific B cells undergo phenotypic changes during chronic HBV infection, the dysfunction of specific antibody secretion and differentiation into plasma cells promotes the persistence of HBV infection and liver damage. TFH plays a key role in the development of antibody-secreting B cells. Unfortunately, both Treg and TFR cells can inhibit the effect of TFH cells on B cells in CHB patients, which consequently hinder the clearance of HBsAg and a resulting functional cure. In addition, although TFH can promote the production of protective neutralizing anti-HBs antibodies by B cells, an excessive number of activated TFH cells can also promote B cells to secret autoantibodies and induce liver inflammation during chronic HBV infection (16). To date, the interaction between TFH cells and B cells in patients with HBV infection remain incompletely understood. It is believed that further in-depth research regarding its molecular role (*e.g.*, the role CD40L, IL-4, and IL-21) will add to the understanding HBV-specific B cell dysfunction.

## Antigen Presentation Function of B Cells

During HBV infection and clearance, HBV-specific T cells are considered to be the main effector cells. The T cell-mediated cellular immune response requires the participation of antigen presenting cells (APCs). B cells, in addition to their function in antibody production, may play a potential role as professional APC during chronic HBV infection (74). According to an early study, HBcAg-specific B cells are roughly 10^5^-fold more efficient than classical non-B cell APCs at presenting HBcAg to both naive Th cells *in vivo* and to T cells *in vitro* (15). This result is consistent with the transcriptional analysis that HBV-specific B cells, in addition to producing antibodies, may present HBV antigen to T cells in an obscure manner (44). Clinically, an increased number of studies have supported the role for B cells as APCs in HBV infection by evaluating the risk of HBV reactivation following rituximab (anti-CD20 antibody) treatment in B cell lymphoma patients (75–77).

In general, professional APCs, including B cells, are able to recognize and bind exogenous antigens, then present these antigens to HBV-specific CD4+ T cells *via* MHC-II to initiate a CD4+ T immune response. Notably, apart from MHC-II molecules, B cells were also found to express relatively high levels of major histocompatibility complex class I (MHC-I) molecules (78). Through the cross-presentation of HBcAg on MHC-I to specific CD8+ T cells, B cells are able to induce an HBcAg-specific cytotoxic T lymphocytes (CTLs) response and further prevent immune tolerance (13). At the same time, HBsAg, as a special exogenous antigen, is also related to the MHC-I molecules expressed on B cells. The study by Barnaba et al. demonstrated that HBsAg-specific B cells can cross-present HBsAg fragments to CTLs through MHC-I molecules (14); however, this process of inducing CTL cytotoxicity can cause the death of HBsAg-specific B cells and hinder the production of protective anti-HBs antibodies, which promote persistent HBV infection and long-term transmission.

During the presentation of the HBV antigen to activate T cells, HBV peptide-MHC complexes on APCs specifically bind the T-cell receptor (TCR) and then initiate the interaction between costimulatory molecules and receptors. Among them, the costimulatory molecules CD80 and CD86 expressed on APCs are required for the differentiation of T cells, and the interaction between CD80/86 and CD28 mediates critical T cell stimulatory signals (79–82). Therefore, phenotypic analysis of the cell surface molecules (especially MHC and CD80/CD86) is used as an effective surrogate readout for the antigen presentation function of B cells (83, 84). However, the frequency of circulating B cells expressing CD86 does not significantly change in CHB patients (85, 86). Notably, the expression of the costimulatory molecule CD80 and the frequency of HBsAg-specific B cells are significantly decreased in patients with immune tolerance, immune activation and immune clearance, but this decrease is reversed in patients after resolution, thus indicating a potentially impaired HBsAg peptide-presenting function of B cells in CHB patients (66). Another costimulatory molecule CD40 expression on B cells is decreased, which might influence the interaction between CD40 and CD40L and the secondary activation signal of B cells in CHB infection (87).

Other APCs such as dendritic cells (DCs), monocytes and macrophages may be also involved in the HBV immune response. DCs, the most efﬁcient professional APCs, have the strongest antigen presenting ability and can stimulate initial T cell activation and proliferation (88). Although most studies have described defects in DCs that can hinder the T cell-mediated response (89–93), some reported functional molecules (e.g., CD83, CD86 and HLA-DR) of *ex vivo*-tested DCs remain largely intact in CHB patients (94, 95). Monocytes (MNs) compose about 10–15% of both the peripheral blood and intrasinusoidal mononuclear cell content. It was reported that MNs were the only professional antigen presenting cell in the peripheral blood of CHB patients to retain an HBsAg depot and *in vitro* differentiation of HBsAg+MNs to DCs stimulated expansion of autologous HBV-specific T cells (96). However, whether MNs from CHB patients might present the HBsAg depot to T cells *in vivo* remains to be determined. Liver macrophages, Kupffer cells (KCs), are the largest macrophage population in the human body. KCs have some credentials as APC, but the balance of data suggests they commonly promote T cell tolerance (97). KCs express MHC-I and MHC-II molecules, as well as co-stimulatory molecules CD80 and CD86 at low density (97). It was reported using a hydrodynamic HBV transfection model that HBcAg triggered TLR-2 on KCs, inducing IL-10 production that suppressed the HBV-specific CD8+T cell response (98). In addition, KCs might induce T cell exhaustion by upregulating galectin-9 expression in CHB patients (99).

Interestingly, in contrast to other APCs, B cells are characterized by the expression of B cell antigen receptors (BCRs), which ensure specificity during the recognition and binding of HBV antigens that mediate primary activation signal of B cells. Second, high affinity BCRs enable B cells to present specific antigens with high efficiency, even at extremely low antigen concentrations (100); in particular, when the host immune response is reactivated, activated, or there are memory B cells expressing MHCII and BCRs exhibiting strong antigen-presenting activity. Specifically, the specific molecular basis by which HBcAg binds to a high frequency of naive B cells might involve a linear motif expressed by some heavy or light BCR chains (101).

Of note, DCs, MNs, and KCs might affect B cells through secreting cytokines or presenting HBV antigen during CHB infection (33, 102–104). Follicular B cells can recognize the HBV antigens that not only exist alone but also are present on the surfaces of DCs or macrophages (102). B cell-activating factor (BAFF), an important cytokine for B lymphocyte activation, is elevated in CHB patients (105, 106). Moreover, HBeAg itself can stimulate monocytes to release BAFF, a response that might be associated with B-cell hyper-activation in CHB patients (104). KCs have been shown to promote the development of Tr1-like cells, which inactivated both Tfh cells and GC B cells *via* secreting IL-10, resulting in impaired GC formation and anti-HBs antibody production (55, 107).

**Figure 2:**
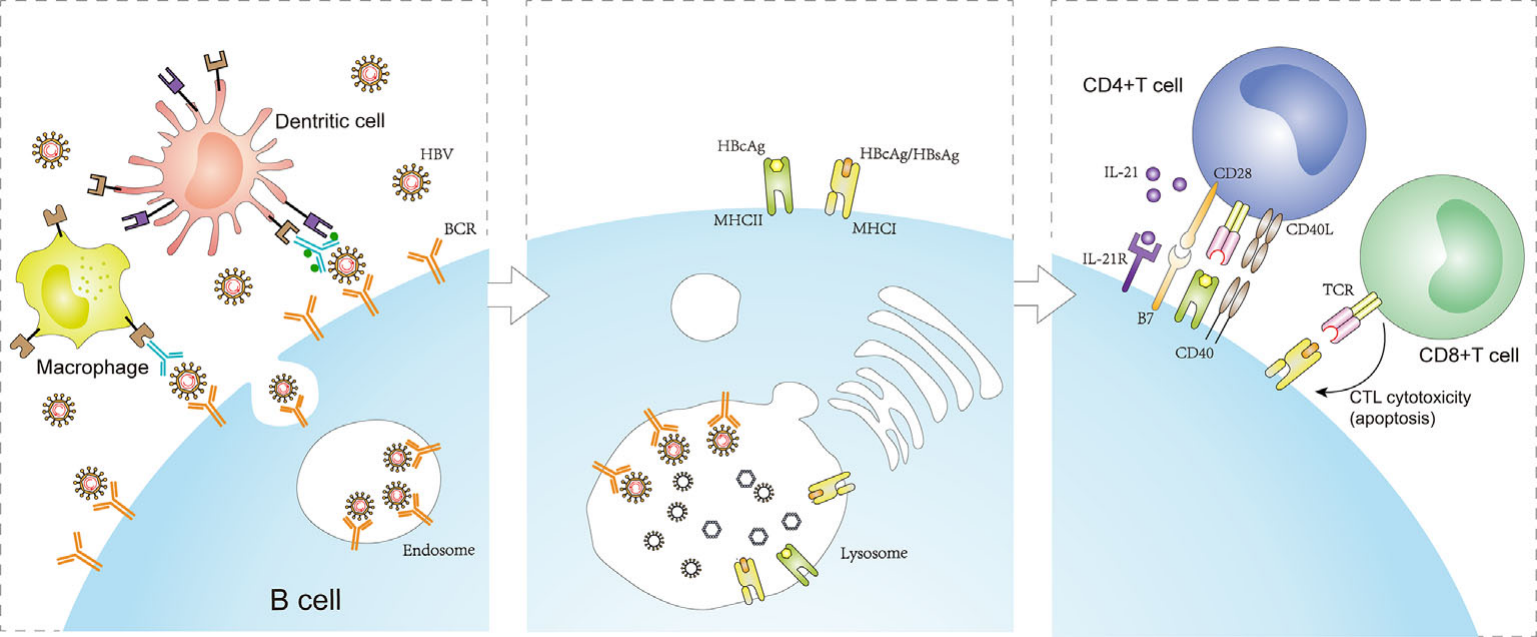
Antigen presentation function of B cells in CHB infection. The BCR specifically recognizes and binds HBV antigens that exist alone or are presented on the surface of macrophages or dendritic cells. The engagement enables BCR-antigen internalization into endosomes. Concomitant with receptor-mediated endocytosis, MHC-I/MHC-II molecules produced by B cells converge to form complexes with the HBcAg/HBsAg peptides processed in lysosomes and transferred to the plasma membrane. Naive CD4+ T cells are activated by B cells presenting HBcAg-MHC-II complexes, inducing a CD4+ T cell immune response. In addition, CD8+ T cells are activated by B cells presenting HBcAg/HBsAg-MHC-I complexes, inducing CTL cytotoxic reaction. However, the process promotes the apoptosis of HBsAg-infected cells (including HBsAg-infected B cells) and persistent HBV infection. BCRs, B cell antigen receptors; MHC-I, major histocompatibility complex class I; MHC-II, major histocompatibility complex class II.

In summary, B cells play an important role in presenting HBcAg to activate a T cell-mediated immune response, which contributes to achieving HBV clearance in chronic HBV infection (15). However, B cells may be attacked by HBsAg-specific CD8+ T cells that are activated by HBsAg presentation, which conversely play a deleterious role in HBV clearance. Regardless, the above immune responses are triggered by the BCR-antigen interaction required for efficient B cell activation (**Figure 2**). Thus, paying attention to the early events in this interaction would help obtain a deeper understanding of the B cell-mediated immune response in CHB patients. Additionally, it is important to note that, compared with healthy populations, a significant decrease or unchanged expression of co-stimulatory molecules, CD80 and CD86, among circulating B cells might be unfavorable for the interaction between B cells and effector T cells, which could not further amplify the T cell response during chronic HBV infection (66, 86). Therefore, there are some limitations associated with B cell presentation of HBV antigen to T cells, which should be also considered during chronic HBV infection.

## Immune Regulatory Function of B Cells

**Figure 3:**
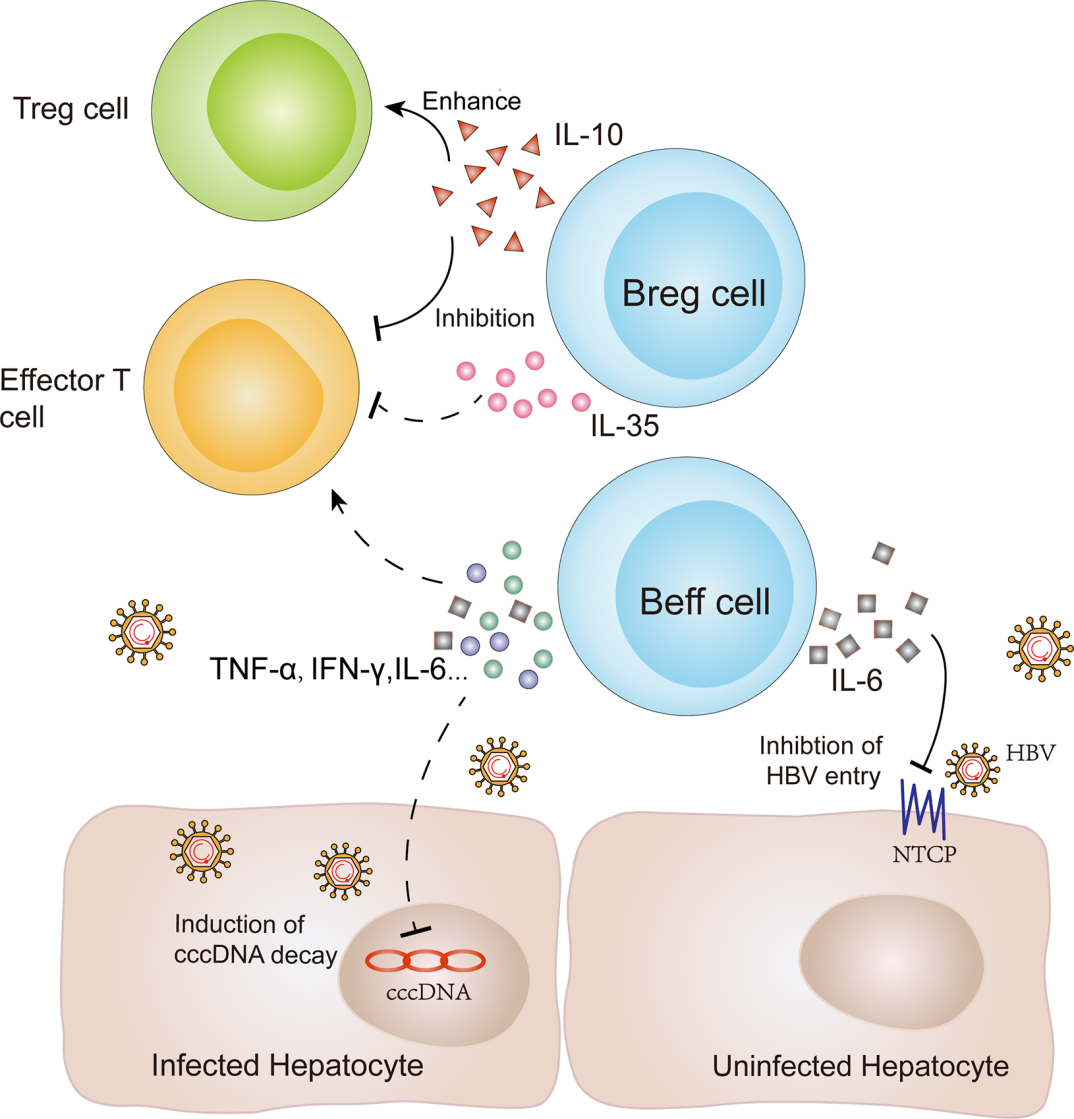
Immune regulation and antiviral function of B cells in CHB infection. B cells play potential immune regulatory roles to induce or inhibit the immune response *via* secreting different cytokines. Breg cells can produce IL-10 to inhibit effector T cell function and enhance Treg cell function. Furthermore, IL-35 secretion by Breg cells can inhibit the proliferation of naive effector T cells. In contrast, Beff cells produce proinflammatory cytokines, including interleukin (IL)-6, interferon (IFN)-*γ*, and tumor necrosis factor (TNF)-α, to promote effector and memory CD4+ T cell responses. At the same time, these cytokines might play a non-cytolytic antiviral role for infected hepatocytes *via* inducing cccDNA decay or reducing HBV transcription. In addition, IL-6 secretion by Beff cells can also inhibit HBV entry by regulating the expression of an HBV-specific receptor, known as NTCP. NTCP: Na(+)/taurocholate co-transporting polypeptide; Breg cells: regulatory B cells; Beff cells: effector B cells.

In addition to antibody production and antigen presentation, B cells also regulate the immune response by secreting cytokines. B cells can be divided into effector B (Beff) cells and regulatory B (Breg) cells according to their specific cytokine secretion profile (108). Among them, Beff cells produce pro-inflammatory cytokines, such as interleukin (IL)-6, interferon (IFN)-*γ* and tumor necrosis factor (TNF)-α, to promote a pro-inflammatory immune response and influence effector and memory CD4+ T cell responses (33, 109). At the same time, these cytokines may play a non-cytolytic antiviral role for infected hepatocytes *via* inducing cccDNA decay or reducing HBV transcription (29, 30, 34). Moreover, two studies have demonstrated that IL-6 can also inhibit HBV entry *via* regulating the expression of an HBV-specific receptor, human liver bile acid transporter Na(+)/taurocholate co-transporting polypeptide (NTCP) (31, 110) (**Figure 3**). However, both the protection of hepatocytes and the tissue injury caused by IL-6 remain controversial and the reduction of these effector cytokines from atMBCs might limit their direct antiviral activity in CHB infection (32).

In contrast to Beff cells, Breg cells are suppressor cells, which secrete IL-10 as a crucial mediator for B cell-mediated regulation of other immune cells and the maintenance of immune tolerance (111). In the human phenotype, IL-10-producing B cells are enriched among CD19+ CD24hi CD38hi transitional B cells (112), CD19+ CD24hi CD27+ B10 cells (113), or both CD27+ memory and CD38hi transitional B cell subsets (114). In addition, Breg cell subsets expressing different surface markers have been identified, including CD19+CD25+ B cells (33), CD5+CD19+CD1dhi IL-10+ B cells (115), CD19+CD5+Foxp3+ B cells (116), and CD25+CD71+CD73− B cells (117). However, a lack of specific markers on Breg cells remains an unresolved challenge and may restrict Breg-related research in HBV infection. In addition, a study by Das et al. found that B cells producing IL-10 primarily exhibited immature/transitional CD19+ CD24hi CD38hi expression in CHB patients (118), which was utilized as a universal marker of Breg cells in related research. In subsequent horizontal and longitudinal studies, it was discovered that the proportion of Breg cells and the level of serum IL-10 were significantly increased and positively correlated with transaminase (ALT and AST) levels, viral load (HBV-DNA), and hepatic flares (118–120). These results suggest that both Breg cells and IL-10 regulate the anti-HBV immune response.

Specifically, IL-10-producing Breg cells play an important role in suppressing the HBV-specific CD8+ T cell response, whereas the depletion of Breg cells might restore the specific CD8+ T cell response (118). Since CD8+ T cells predominantly mediate the anti-HBV immune response, it can be inferred that both Breg cells and IL-10 are involved in HBV immune tolerance through suppressing CD8+ T cells during chronic HBV infection. However, the immune regulation of Breg cells is reflected by both the inhibition of CD8+ T cells, as well as the regulation of the CD4+ T cell response. Moreover, IL-10+ Breg cells can promote the inflammatory response of CD4+ CD25− effector T cells and the conversion to CD4+ CD25+ Treg cells, which suppress CTLs (25). Thus, Breg cells play a crucial role in inducing HBV immune tolerance through inhibiting effector T cells (effector CD4+ and CD8+ T cells) and enhancing Treg cells.

Although Breg cells regulate the Treg cell response, Treg cells have fewer effects on Breg cells during chronic HBV infection (26). However, TFR cells, which exhibit a similar inhibitory capacity as Treg cells, might influence the function of Breg cells. Wang et al. demonstrated that cTFR cells were enriched in the peripheral blood and elevated the inhibitory effect of Breg cells on CD8+ T cells *in vitro* (64). Furthermore, the signaling molecules associated with the regulatory function of Breg cells remain obscure during chronic HBV infection, in which Toll-like receptors (TLRs), B cell receptor (BCR), CD40, and costimulatory molecules CD80-CD86 on Breg cells may be considered to be relevant factors (121).

As described above, Beff cells produce proinflammatory cytokines to promote an HBV-specific immune response, whereas Breg cells play an immunosuppressive role with the help of cTFR cells in chronic HBV infection. Among them, IL-10 produced by Breg cells is a crucial cytokine associated with immune regulation. In addition, it is important to note that IL-35 is produced by Breg and Treg cells, as a novel member of the IL-12 family, which may represent another suppressive cytokine (27). Li et al. found that high levels of IL-35 expression in CD4+ T cells could inhibit the proliferation of HBV-specific CTL cells and the production of IFN-gamma (IFN-*γ*) *in vitro* (28). However, there remains a lack of research on IL-35 derived from Breg cells in HBV infection.

## Immunotherapeutic Prospects of B Cells in Chronic HBV Infection

In lymphoma patients with HBV infection, treatment with rituximab (a therapeutic monoclonal antibody against CD20 that induces a profound depletion of B cells) significantly increases HBV reactivation (77, 122). This indicates that causing an effective B cell response (*e.g.*, proliferation, cytokine secretion, and specific antibody response) and maintaining a stable B cell state are essential for the prevention of HBV reactivation and clearance of an HBV infection. Currently, pegylated interferon (Peg-IFN) and nucleoside analogs (NAs) have been approved and recommended as common first-line antiviral therapies for chronic HBV infection (123). In addition, these antiviral treatments are commonly used in two of the four clinical stages of chronic HBV infection: 1) immune activity (HBeAg-positive hepatitis); and 2) HBeAg negative hepatitis. In almost all patients with chronic HBV infection, these therapies display high efficacy, including disturbed B cell homeostasis, can be partially recovered (124); however, difficulties remain in achieving a loss of HBsAg and functional cure (persistently undetectable HBsAg) (125). A high load of HBsAg could further inhibit adaptive immune function and ultimately leads to specific tolerance that prevents patients from eradicating HBV infection. In addition, lifelong requirements for HBV antiviral therapy may increase the risk of drug toxicity. Under such circumstances, the recovery of B cell hyperactivation and functional impairment can contribute to HBsAg seroconversion of CHB patients (66). Thus, the development of long-term successful immunotherapy to further inhibit HBsAg *via* utilizing the multiple functions of B cells is required (**Table 2**).

**Table 2:** Current immunotherapeutic approaches related to the multiple functions of B cells.

Immunotherapeutic approaches		Therapeutics	Status	Therapeutic effect	References
**Therapeutic vaccination**	preS1-polypeptide	preS1 vaccine	Animal	Induced robust humoral responses in HBV carrier mice.	(126)
	HBsAg + HBcAg	NASVAC	Clinical III	Acted as a safe and efficient therapeutic approach in CHB patients.	(127, 128)
	X, large-S	GS4774	Clinical I/II	Safe and well tolerated in healthy subjects, while no significant HBsAg reductions in virally suppressed CHB patients.	(129, 130)
	PreS2+S	DNA vaccine	Clinical I/II	No great effect on immunotolerant CHB patients or inactive HBsAg carriers.	(131–133)
	PreS1+PreS2+S	DNA vaccine	Clinical	Induced anti-HBs antibodies in around half HBeAg+ CHB patients.	(134)
	S+PreS1+Core	DNA vaccine	Animal	Induced robust and durable humoral responses against PreS1/HBsAg/HBcAg in rhesus macaques.	(135)
	HBsAg-aa119-125	CR-T3-SEQ13	Animal	Induced a pivotal and long-term anti-HBs antibody response in HBV-tolerant mice.	(136)
**Checkpoint inhibitor**	Anti-PD-1	Nivolumab	Clinical I	Partially rescued HBsAg-specific B cells function *in vitro* through anti-PD-1. Meanwhile, safe and effective for viral suppressed CHB patients.	(42, 137)
**TLR agonist**	TLR-7 agonist	GS-9620	Clinical II	Safe and well-tolerated in particular CHB patients, while no significant HBsAg reductions in virally suppressed CHB patients.	(138–140)
	TLR-9 agonist	CPG 7909	Clinical I	Antigen-specific, isotype-specific and induced high affinity antibodies.	(141–143)
		1018 ISS	Clinical	Well-tolerated in healthy adults, and induced a high antibody response when co-administrated with HBsAg.	(144, 145)
**Therapeutic cell adjuvant**	sCD40L-activated B cells	B cells adjuvants	Vitro	Acted as APCs and induced HBV-specific CTL immune response *in vitro*.	(146)

## Therapeutic HBV Vaccines

In animal experiments and early clinical studies, therapeutic vaccinations which trigger a novel immune response have substantial application prospects. To date, several categories of immunogens have been developed, including protein- or polypeptide-based, DNA- and viral vector-based vaccines. A vaccination with long peptide of preS1 domain was explored to clear HBV virions. Anti-preS1 antibody produced through sequential administration of preS1/HBsAg vaccines could finally induce HBsAg seroconversion in HBV carrier mice (126). In C57BL/6 mice, a therapeutic vaccine consisting of HBsAg/HBcAg and the CpG adjuvant elicited forceful humoral responses (147). When combined with saponin-based adjuvants, HBcAg/HBsAg compound vaccines could also induce cellular and humoral immune responses in HBV transgenic mice (148). The HBsAg-HBcAg nasal vaccine candidate (NASVAC) has shown safety and high immunogenicity in a phase I clinical trial (127, 149). Even in an open phase III trial, the efficacy of NASVAC in anti-HBV and HBeAg clearance is comparable to Peg-IFN in CHB patients (128). The recombinant yeast-based vaccine, GS-4774, contains HBV-speciﬁc antigens such as HBx protein and large HBsAg. Its safety, tolerability and immunogenicity have been veriﬁed in healthy participants (129). However, GS-4774 showed no beneﬁt in significant HBsAg reduction in virally suppressed CHB patients (130).

Some DNA vaccines, such as PreS2 +S or PreS1-PreS2-S, also have been developed and found to induce an HBV-specific immune response in clinical trials (131, 134). In two randomized-controlled studies, PreS2 +S had limited therapeutic efficacy in immunotolerant CHB patients or inactive HBsAg carriers (132, 133). In contrast, the PreS1-PreS2-S vaccine induced anti-HBs antibodies in approximately half of the HBeAg+ CHB patients (134). Recently, novel DNA vaccine candidates comprising the S, PreS1, and Core antigens has been shown to induce specific immune response in rhesus macaques (135).

However, most therapeutic vaccinations depend on inducing an effective T cell response instead of B cell and antibody responses (130, 131). Moreover, there are limitations associated with completely restoring T cell functions and removing HBsAg with vaccinations (130, 132–134). Recently, based on the specific antibody-recognized HBsAg epitope (HBsAg-aa119−125), Zhang et al. developed a vaccine candidate (CR-T3-SEQ13) which induced a pivotal and long-term anti-HBs antibody response (136). Although studies related to the CR-T3-SEQ13 vaccine candidate are only in preliminary animal models, the therapeutic efficacy for improving the B cell-targeting vaccines might be expected.

Notably, the distributions of HBV genotypes and HLA alleles differ worldwide (150, 151). Developing therapeutic vaccines targeting B cells requires a sufficiently broad B cell repertoire covering the conserved regions of the HBV virus to ensure efficacy for different HLA populations and to prevent viral escape. In addition, most therapeutic vaccines are not developed primarily to restore the B cell response, and the detection of their functions are often neglected. Further exploring the changes in B cell function after treatment with vaccines might contribute to optimization of vaccine therapeutic approaches. Ultimately, clinical trials using HBsAg-based therapeutic vaccination have not shown lasting therapeutic effects in patients after cessation of nucleos(t)ide analogue treatment (152). Therefore, a combination of different immunotherapeutic interventions might be necessary to induce the B cell antibody response and HBV elimination.

## TLR Agonists and Checkpoint Inhibitors

In contrast to therapeutic vaccinations, treatment with TLR agonists and checkpoint inhibitors is designed to reinvigorate the function of pre-existing anti-HBV immunity. Suthers et al. reported that crosstalk between the TLR7/TLR9 signaling pathway and the B cell receptor (BCR) may regulate the B cell response to antigens (153). Compared to antigen alone, mice that received both the antigen and TLR7/TLR9 ligand induced a more robust antibody response (154, 155).The TLR7 agonist GS-9620 has been shown to induce sustained humoral responses in woodchucks and chimps with CHB infection (156, 157). In addition, its safety and tolerance have been demonstrated in some clinical studies (138, 139). The TLR9 agonists CPG 7909 or 1018 ISS, co-administrated with HBsAg, have been found to induce a high antibody response in CHB patients (141–145). These results indicate that TLR7/TLR9 agonists may serve as adjuvants to improve the efficacy of B cell-targeting vaccines. The PD-1 checkpoint inhibitor, nivolumab, has been found to be safe and to lead to a decline in HBsAg in most virally suppressed CHB patients in a phase I clinical trial (137). Of note, the dysfunction of anti-HBs antibody-secreting B cells can be partially recovered by blocking PD-1, supporting the use of a PD-1 inhibitor to restore the normal function of B cells outside of T cells (42).

Furthermore, the expression of not only PD-1, but also the inhibitory receptors FcRL5, FcyRIIB, BTLA and CD22 is consistently enriched on HBsAg-specific B cells (32). Given the inhibition of BCR signaling, FcRL5 and Fc*γ*RIIB are likely to represent novel B cell therapeutic targets (158–160).

## Activated B Cell Adjuvants

Currently, it is difficult to completely recover T cell function through clinical treatment, which also hinders the eradication of HBV infection. Therefore, in addition to B cell secretion of anti-HBs antibodies, attention should also be paid to the effect of B cells on T cells. To induce an HBV-specific T cell response, HBsAg/HBcAg-pulsed DC-vaccines have been designed to play the role of APCs and have exhibited immunogenicity in a phase I clinical trial (161). However, the high cost and associated technical requirements restrict the clinical application of DC adjuvants. At this point, utilizing B cells as APCs to present antigens may represent an alternative strategy to induce T cell-mediated immunity. Wu et al. demonstrated that the expression of MHC-I, MHC-II, CD80, and CD86 on B cells was increased following stimulation with human soluble CD40L (sCD40L) *in vitro*, and activated B cells may represent an adjuvant to present HBcAg to CTLs (146).

## Target Breg Cells

In contrast to antigen presentation, B cells play an adverse role through the secretion of IL-10 during chronic HBV infection. As previously mentioned, Breg cells can both inhibit the effector T cell response, as well as enhance Treg cell suppression to further promote T cell-mediated immune tolerance. Therefore, it is possible to induce an effective immune response *via* effective Breg cell-targeted therapy in the future (162). However, treatment approaches that target Breg cells in chronic HBV infection remain lacking, one of the major limiting factors is the lack of surface-specific Breg cell markers (162). In HCC studies, enhanced expression of PD-1 has been found in a unique Breg cell subset (163); PD-1 blockade can be utilized to target Breg cells (164), indicating the importance of exploring specific Breg cell markers in CHB patients. In studies investigating the treatment of HCV infection and HCC, researchers have constructed the recombinant plasmid pcCD19scFv-IL10R to target mouse B10 cells and enhance the anti-HCV immune response or inhibit HCC growth *in vivo* (165, 166). These findings might also provide some clues for targeting Breg cells during CHB infection. Furthermore, it is important to note that blocking the inhibitory cytokines produced by Breg cells (*e.g.*, IL-10 and IL-35) may also be useful to suppress the harmful effects of Breg cells and restore the effector T cell response in CHB patients (167).

In summary, targeting Breg cells is a promising immune therapy for CHB infection. However, some issues should be considered when developing approaches to target Breg cells. Although excessive Breg cells may lead to HBV immune tolerance, suitable Breg cells may contribute to suppressing HBV inflammation. The therapeutic degree of targeting B cells must be further investigated.

## Conclusions and Future Research Agenda

HBV infection disrupts B cell homeostasis, leading to the dysregulation of B cell function (**Table 3**). Abnormal B cells play a significant role in a persistent HBV infection, as well as T cell-mediated immune tolerance and antibody-associated liver damage. Among these, antibody secretion, as well as immune regulation and antigen presentation of B cells are important in the pathogenesis of HBV infection.

**Table 3:** The role of B cell subsets in chronic HBV infection.

Subset	Molecular phenotype	The roles in CHB infection	References
**Naive B cells**	CD10^-^ CD19^+^ CD27^−^ IgD^+^	Present HBcAg/HBsAg on MHC-I/MHC-II to specific T cells during early HBV infection	(13–15, 74)
**classical memory B cells (cMBCs)**	CD19^+^ CD20^+^ CD27^+^ CD21^+^	1. Quickly differentiate into plasma cells and produce antibodies in HBV re-infection 2. Present HBV antigen to activate T cells 3. Secret cytokines to regulate immune response	(32, 74)
**atypical memory B cells (atMBCs)**	CD19^+^ CD20^+^ CD27^−^ CD21^–^	Defect in antiviral cytokine production (*e.g.*, IL-6, TNF-α) and differentiation into antibody-producing cells	(32)
**HBcAg specific Bm cells**	CD19^+^ CD20^+^ CD21^+^ CD27^+^ CD24^hi^ (similar with cMBCs)	1. Expand and mature into plasma cells to produce anti-HBc antibodies during HBV re-infection 2. Present HBcAg on MHC-I/MHC-II to activate specific T cells	(13, 15, 44, 74)
**HBsAg specific Bm cells**	CD19^+^ CD20^+^ CD21^−^ CD27^−^ PD1^hi^ FcRL5^hi^ (similar with atMBCs)	1. Defect in the differentiation of plasma cells to produce anti-HBs antibodies in HBV re-infection 2. Cross-present HBsAg on MHC-I to activate specific CD8+ T cells	(14, 32, 42, 44, 74)
**Plasma cells**	CD19^+^ CD20^-^ CD27^+^ CD38^hi^ CD138^+^	Produce long-lasting IgG antibodies (e.g., anti-HBsAg antibodies)	(65, 66)
**Plasmablasts**	CD19^+^ CD24^hi^ CD38^+^ CD27^−^ CD20^-/lo^	Defect on differentiating into plasma cells and producing IgG antibodies in early HBV infection	(64)
**Immature/transitional B cells**	CD19^+^ CD24^hi^ CD38^hi^ (the main represent of IL-10+B cells)	Suppress T eﬀector cells but promote Treg cells by producing IL-10	(25, 118)
**Beff cells**	CD11a^hi^ FcγRIII^hi^ (lack of specific markers)	Defect in the secretion of antiviral effector cytokines (*e.g*., IL-6 and TNF-α)	(29–33, 109)

It is important to note that B cell functionality is multi-dimensional. Specifically, in a highly immunogenic HBcAg infection, B cell-mediated cross-presentation may induce CTLs (13, 15), while Breg cells suppress the CTL response (118). In persistent HBsAg infection, B cells may secrete neutralizing antibodies to play a beneficial role (20, 21); however, B cells may also cross-present HBsAg to CTL cells to play an adverse role (14). Therefore, it would meaningful to elucidate how to restore B cell homeostasis and what measures should be taken in consideration of the advantages and disadvantages of B cells in HBV treatment.

In addition, particular attention should be paid to the effect of T cells on B cells, in which the dysfunction of TFH cells caused by Tregs and TFRs hinders the ability of B cells to secrete anti-HBs antibodies (52, 54–56). The development of HBV vaccines to restore the role of TFH cells may also facilitate B cell-mediated humoral immunity and the production of anti-HBs antibodies to functionally cure CHB patients.

Finally, in this paper, we primarily discuss the B cell function in adults with chronic HBV infection. In newborns, many components of the immune system are underdeveloped, and defects in the humoral and cellular immune response contribute to a predisposition to infection (168, 169). Based on B cell phenotype detection, HBsAg-positive infants had a higher level of immature transitional B cells at birth, which returned to normal one year after receiving the hepatitis B vaccine (170). These findings highlight the defects of B cells in neonatal HBV infection and the importance in developing vaccines to induce B cell maturation. In children with chronic hepatitis B infection, ANA(antinuclear antibodies) formation and a lower percentage of CD19+ B lymphocytes is part of the natural course of chronic infection, which may reflect a tendency towards autoimmune disease (171). Therefore, the functional characteristics of B cells in infants should be explored to optimize vaccine formulations for the early prevention and treatment of HBV infection in infants.

## Author Contributions

WY designed and supervised the manuscript. YC and WY prepared the manuscript. All authors contributed to the article and approved the submitted version.

## Funding

This study was funded by the National Natural Science Foundation of China (81770570, 81801990) and the Program for Outstanding Young Talent of Chongqing Kuanren Hospital and Natural Science Foundation of Chongqing, China (cstc2019jcyj-msxmX0007).

## Conflict of Interest

The authors declare that the research was conducted in the absence of any commercial or financial relationships that could be construed as a potential conflict of interest.
